# External resilience in the context of drug use and socio-structural vulnerabilities: a qualitative exploration among women who use drugs and sell sex in Baltimore, Maryland

**DOI:** 10.1186/s12954-022-00678-6

**Published:** 2022-08-24

**Authors:** Catherine Tomko, Danielle Friedman Nestadt, Noelle P. Weicker, Katherine Rudzinski, Carol Underwood, Michelle R. Kaufman, Susan G. Sherman

**Affiliations:** 1 Department of Mental Health, Johns Hopkins Bloomberg School of Public Health, 624 N. Broadway, Baltimore, MD 21205 USA; 2 Department of Health, Behavior and Society, Johns Hopkins Bloomberg School of Public Health, 624 N. Broadway, Baltimore, MD 21205 USA; 3 Division of Social and Behavioral Health Sciences, Dalla Lana School of Public Health, University of Toronto, 155 College Street, 6th Floor, Toronto, ON M5T 3M7 Canada

**Keywords:** Resilience, Social support, Substance use, Women, Sex work

## Abstract

**Background:**

Resilience is a commonly used construct in substance use and mental health research. Yet it is often narrowly defined by only its internal qualities (e.g., adaptability, hardiness) and overlooks its external qualities (e.g., supportive relationships, navigating one’s environment). Further, substance use is often viewed as antithetical to resilience despite populations like women who use drugs and sell sex (WWUD-SS) surviving significant hardships. This study aims to fill a gap in the literature by characterizing external resilience among WWUD-SS and understanding the ways that socio-structural vulnerabilities (e.g., poverty, stigma) and substance use shape external resilience.

**Methods:**

WWUD-SS (*N* = 18) enrolled in an ongoing cohort study were purposively sampled for age, race, and recruitment location and participated in semi-structured, in-depth interviews aimed to elucidate external resilience (i.e., social support and resource utilization). WWUD-SS were queried about recent difficult experiences with a focus on how they did or did not use social support or formal resources (e.g., clinic, crisis hotline) in response.

**Results:**

Participants were a median age of 37 years, 50% identified as Black, and 50% reported currently injecting drugs. Participants described reluctance to ask for support and often felt resigned to address problems alone. Participants also distinguished between transactional relationships (help is contingent upon receiving something in return) versus genuine (non-transactional or altruistic) support, including the role of family members who do and do not use drugs. Resource utilization was rare, and “self-medication” through substance use was common absent other perceived options for help.

**Conclusions:**

External resilience appears limited among WWUD-SS and shaped by the social and economic contexts of a street-involved life. WWUD-SS’ ability to exercise external resilience may be undercut by experiencing structural vulnerabilities and competition for material resources that create transactional relationships and diminish the perceived value of social support. Internalized stigma, reflecting the larger society’s stigmatized views of drug use, sex work, and poverty, left WWUD-SS eschewing help from outside sources. Focus on internal resilience alone offers an incomplete picture of the construct in drug-using populations. Improving connections to community resources may be a targeted way to strengthen external resilience, as are policies addressing structural vulnerabilities for marginalized communities.

## Introduction

Globally, women who use drugs and sell sex (WWUD-SS) bear a high mental health burden, that includes rates of depression (estimated at 29–82%) and post-traumatic stress disorder (PTSD, estimated at 13–68%) much higher than the general female population, and significant exposure to chronic and severe stressors such as violence and stigma that can heighten psychological distress [1–8]. For WWUD-SS, economic precarity is common, as is the threat of arrest and incarceration as a result of the criminalization of sex work and controlled substances, particularly in the USA [9–11]. These exposures, independently and synergistically, heighten the risk for mental distress, overdose, and HIV acquisition [2–4, 12–15].

Despite this disparity between WWUD-SS and the general population, little research has focused on modifiable factors to improve mental health in this population, such as resilience. Definitions of resilience and prevailing resilience theory have evolved over time, from resilience as a fixed personality trait to the current conceptualization of resilience as a dynamic, multisystem process [16]. Broadly, resilience is “the achievement of positive adaptation” in the face of “significant” threats or adversities [17, 18]. However, as a dynamic, multisystem process, resilience may manifest in multiple pathways and across multiple levels of the socio-ecological model, with changes through developmental stages and throughout the life course.

Current resilience theory recognizes sources of resilience both within and external to an individual. A review of existing resilience scales confirms this dichotomy of internal and external dimensions of resilience [19]. Internal resilience factors are qualities related to the person, such as adaptability, self-efficacy, active coping, positive emotions, mastery, and hardiness. External resilience factors are outside of the individual and related to a situation, such as supportive relationships, planning and organizing abilities within the environment, and accessing community-based resources [19]. Pangallo et al. describe resilience from a dynamic interactionism perspective, one where internal and external resilience are not shaped separately but mutually shape and reinforce each other [19]. This dynamic interactionism of resilience reflects the fundamental tension between the degree to which structures (e.g., social, political) impose constraints on individuals’ behaviors while individuals also exert agency over that structure [20]. Drawing upon sources of external resilience may enhance feelings of internal resilience, strengthening a sense of agency over accessing sources of external resilience in the future. However, for WWUD-SS, chronic and frequent stressors contribute to physical, cognitive, and emotional fatigue or distress that can lead to compromised ability to exert agency to harness external resilience [21, 22].

Multi-level sources of stigma are pervasive in the lives of WWUD-SS and may play an important role in the dynamic interactionism of resilience. The social stigma attached to drug use or sex work serves as a barrier to help-seeking, social support, or other forms of external resilience. Internalized stigma is a common barrier to external resilience, including by preventing WWUD-SS from deciding to access health care or other services or by undercutting social support via shame or guilt about disclosing these behaviors to social networks [23–27]. Structural stigma similarly affects external resilience through the implicit exclusion of WWUD-SS from forms of help-seeking. For example, WWUD-SS often cite fear of criminal legal consequences stemming from the criminalization of sex work and drug use as a barrier to seeking out health care [24, 28, 29]. Perceived and enacted stigma-related barriers to external resilience can also influence diminished feelings of internal resilience by creating a sense of helplessness, lack of individual agency, or isolation from others—either because women felt they could not ask for help, or had poor past experiences with those from whom they sought help. Collectively, these feelings can produce “limited trust in response systems,” or a lack of confidence in receiving supportive, helpful, and affirming care, thereby threatening women’s external resilience [30].

Resilience is an important psychosocial construct that has consistently been associated with better mental health outcomes, such as decreased symptoms of depression and anxiety, and positively correlated with markers of improved psychological functioning (e.g., life satisfaction), though these findings have nearly exclusively considered internal resilience [31]. Social support is one dimension of external resilience and has been shown to have positive associations with mental health outcomes in WWUD-SS [32–37]. Based on research conducted on samples of people who use drugs and those who do not, the utility and salience of social support may be greater for women compared to men [38, 39]. Receiving social support has more consistently buffered feelings of psychological distress after stressors or trauma for women than men [40, 41]. Community empowerment interventions, which have been implemented and evaluated among women who sell sex in many countries, are one type of approach to building external resilience: Their goal is to build collective agency among the community to address disempowering social contexts that promote risk, with social support a crucial part of that agency-building [42]. However, women whose social networks provide inadequate or untimely support may subsequently feel more distress than men in similar circumstances; this gendered difference in the importance of quality social support may be one reason why women often experience greater feelings of psychological distress [39, 41]. For women, social support often comes from romantic partners—many of whom are men—though experiencing violence from partners that use drugs is common and can undermine social connectedness [43, 44]. For these reasons, it is particularly important to understand social support as a form of external resilience in a woman population such as WWUD-SS that experience frequent stressors and psychological distress.

To date, few studies have examined resilience among WWUD-SS in North America and have nearly exclusively focused on internal resilience [34, 35, 45, 46]. Nevertheless, these studies have found that internal resilience can be a counterbalance to discrimination and stigmatization from others but that structural vulnerabilities, such as housing, food insecurities, and violence, play a central role in diminishing resilience [34, 46]. For people who use drugs, resilience is typically positioned as antithetical to substance use. In a scoping review of studies of resilience among people who use drugs, Rudzinski et al*.* found that substance use was often framed as “maladaptive” and as an outcome synonymous with a lack of resilience. Further, ceasing substance use was also framed as a behavior indicating greater resilience, leaving little room for understanding of resilience among people who actively use drugs [47]. A small body of literature has examined WWUD-SS’ barriers to utilizing resources such as primary and emergency health care and HIV or sexually transmitted infection testing and treatment [48]. None of these studies, however, purported to study these domains specifically in the context of harnessing external resilience factors to overcome hardship.

In this qualitative study, we aimed to present a more comprehensive picture of WWUD-SS’ external resilience by describing key themes related to social support and resource utilization after experiencing hardships and traumatic events.

## Materials and methods

### Recruitment and data collection

The quantitative data for this study were collected between September 2017 and February 2019 as part of a larger parent study (EMERALD) of cisgender women sex workers (*N* = 385) in Baltimore. A detailed description of recruitment and survey data collection protocols was published elsewhere [49]. Briefly, EMERALD was a non-randomized, community-based structural intervention consisting of a drop-in center serving the health and social service needs of women-identified clients who sell sex and/or use drugs. Geographic areas in Baltimore immediately adjacent to the drop-in center were considered part of the intervention; other recruitment areas not adjacent to the drop-in center were control areas. Participants completed surveys every 6 months for 18 months. Eligibility criteria included: (1) aged 18 or older; (2) cisgender woman; (3) sold or traded oral, vaginal, or anal sex at least three times in the past 3 months. Baseline demographic and other key descriptive characteristics presented in this manuscript were collected in the parent study, including age; race; length of time in sex work; substance use history (i.e., daily opioid use, daily drug injection, and daily crack cocaine use); and structural vulnerabilities (i.e., experiencing homelessness, weekly food insecurity, and financial dependence on another person).

We recruited qualitative sub-study participants from the EMERALD cohort in June 2019. All EMERALD study participants were eligible for qualitative interviews. Three days a week, a qualitative interviewer was present during standard survey data collection shifts. During these times, the study staff asked participants if they were interested in completing an interview and provided some basic information about the sub-study. Those who were interested were referred to the interviewer on site. The subsample was recruited through a purposive sampling strategy in order to have an equal number in the intervention and control areas as well as roughly mirror the cohort in age and race/ethnicity. We initially recruited ten participants (half from intervention areas, half from control) and recorded their age and race. After this, we alternated recruitment from intervention and control areas and asked study staff recruiting participants to first consider race and age range according to sampling needs.

After obtaining verbal informed consent from participants, three experienced qualitative interviewers used in-depth, semi-structured interview guides to engage participants in one-on-one discussions about external dimensions of resilience. Interviewers were encouraged to probe where needed and to make the interview conversational. The interview guide briefly covered participants’ day-to-day life, and then participants were encouraged to tell a story about a difficult time they recently experienced; there were no limitations on what participants defined as a “difficult experience.” From there, questions related to social support and formal resource use were asked using their story as the context. Interviewers were trained on which parts of the interview guides were essential to cover and which were less important. Interviewers were also reminded that interviews did not need to be linear in service of a conversational tone. Interviews were conducted in a private area of the mobile van (if space was available) or in the interviewer’s car, in view of the EMERALD staff for safety. All interviews were audio-recorded and transcribed verbatim. Participants were given a $25 VISA gift card for remuneration for the qualitative sub-study. The study was approved by the Institutional Review Board at the Johns Hopkins Bloomberg School of Public Health.

### Analysis

The target sample size (*N* = 25) was determined by balancing code saturation (i.e., determining enough codes so that researchers have “heard it all”) and meaning saturation (i.e., having enough data to “understand it all”), though the lead author determined meaning and code saturation were both met at 18 interviews, at which time data collection ceased [50]. The lead author read each transcript and developed the initial coding scheme based on a mixed inductive and deductive approach. This coding structure was then applied to a set of two transcripts where two coders independently coded interviews and met to discuss code applications and any new codes that emerged. Inter-coder reliability was reached when both coders reached a consensus on the code applications after discussion. Codes were finalized, and two team members coded the remaining interviews. To begin an analysis, we used the one sheet of paper (OSOP) technique for the broadest codes (e.g., social support) to reveal emergent patterns and themes [51]. OSOP involves reading through all segments from a code and writing commonalities on a sheet of paper, grouped by narrower pattern and theme. Then, themes and codes were compared and discussed among the authors to reveal overarching themes in the data. Participants were given pseudonyms throughout this manuscript to preserve anonymity. MAXQDA (VERBI Software, Berlin) was used for all data management and analysis.

## Results

Participants were a median of 37 years old (range 19–62) and had spent a median of 13.5 years (range 1–30) in sex work (Table 1). Eight participants were non-Hispanic white, nine were non-Hispanic Black, and one participant responded “other” race but did not clarify. Half of the participants (*n* = 9) currently injected drugs, most commonly heroin or speedball (i.e., heroin and cocaine together). Interviews were an average of 30 min long (range 17–50 min). Because our analysis did not identify any patterns in differences between participants recruited from intervention and control areas, we have not included that as part of the descriptions of each participant below.

**Table 1:** Characteristics of qualitative interview participants, a subsample of the EMERALD cohort of female sex workers in Baltimore, Maryland (*n* = 18)

“Name”	Age	Race	Years in sex work	Drug use history			Structural vulnerabilities		
				Daily opioid use	Daily drug injection	Daily crack cocaine use	Experiencing homelessness	Food insecure	Financial dependence on another person
Ann	38	NH White	15	No	No	No	No	Yes	No
Beth	19	NH Black	5	Yes	Yes	No	Yes	Yes	No
Carly	44	NH Black	26	No	No	No	No	No	Yes
Deborah	59	NH White	34	Yes	Yes	Yes	Yes	Yes	No
Eve	43	NH Black	27	No	No	No	No	No	No
Frida	23	NH White	12	Yes	Yes	Yes	Yes	Yes	Yes
Georgia	35	NH White	9	Yes	Yes	Yes	No	Yes	Yes
Helen	20	NH White	< 1 year	Yes	Yes	Yes	Yes	Yes	Yes
Irene	41	NH White	24	No	No	Yes	Yes	Yes	No
Jane	31	NH Black	15	No	No	Yes	Yes	Yes	Yes
Kylie	26	Other	4	Yes	Yes	Yes	Yes	Yes	Yes
Lydia	29	NH White	11	Yes	Yes	Yes	Yes	Yes	Yes
Marie	32	NH White	6	Yes	Yes	No	No	No	Yes
Nia	30	NH Black	2	No	No	No	Yes	Yes	Yes
Olivia	43	NH Black	30	Yes	No	Yes	Yes	Yes	Yes
Pearl	46	NH Black	22	Yes	Yes	Yes	No	No	No
Ruth	62	NH Black	12	Yes	No	No	No	No	Yes
Sarah	48	NH Black	18	No	No	No	No	No	Yes

All names are pseudonyms

*NH* = non-Hispanic

In response to the prompt about recent difficult times they faced, participants described a wide variety of traumas and hardships. Some described difficulties that stemmed from their involvement in sex work, drug use, or the drug trade such as sexual assault by paying clients, robbery, and drug overdose. Others described struggles in their personal lives such as the death of a loved one or the breakup of an intimate relationship. Participants described traumatic events throughout the course of the interview outside of the explicit prompt, speaking to the ubiquity of trauma in many of their lives.

### Reluctance to ask for support

For nearly every woman, sex work was influenced by the economic insecurity associated with a current or former history of drug use. Relatedly, asking for help was not an option because women perceived themselves as responsible for their own troubles, perceived to be rooted in drug use and sex work. This meant that sex work and drug use stigmas were often intersecting and mutually reinforcing: If the participant did not need to buy drugs, she would not have had to sell sex, which meant that violence perpetrated against her was of her own making and therefore not worthy of assistance from others. When asked what she did after being assaulted by several people for refusing to have sex with a man, Helen (20 years old) responded, “Nothin’. Just took it [*laughs*]. Just took the ass-beating…. I guess you could say that’s what comes with this [life].”

Some women said that they wanted support from others but found it difficult to seek or accept help. Some described these challenges grounded in having to be self-sufficient early on after growing up with parents who abused substances:

I'm used to doing everything on my own. Since I was 15. … When I was 12 and all that, I was raising my brothers. I'd never see her. My mother was doing drugs, and I just had to do everything in the home. (Jane, 31 years old)

### Transactional versus genuine support

For women, not all forms of support were equal in value. When asked to describe a typical day, women often described being surrounded by others: acquaintances, neighbors, family, other WWUD-SS, or “friends.” When questioned about recent social support, participants drew stark contrasts between people who provided transactional support (i.e., they want or expect something in return for their help) and people who provide genuine, non-transactional support.

Sources of transactional support were much more widely available, but women were hesitant to use these, if at all. People providing transactional support, which sometimes included family members or romantic partners, were best characterized by participant Helen (20 years old) as “people out here,” meaning other peers in the drug and sex work economies. Competition for money and drugs was often seen as the factor undermining social cohesion and support among WWUD-SS: “Money, drugs, foods, and all that. They help each other, and they fight with each other, basically, [*laughs*] over money and drugs, too” (Nia, 30 years old). With money and drugs in scarce supply, participants felt as though anything another woman gets takes away from her own supply.

Help was readily available from these sources for small amounts of money or food, tips about dangerous clients, or even drugs to stave off withdrawal. But receiving help from these people always came with explicit demands for something in return. When asked about approaching someone else to talk about a problem, Ann [38 years old] said: “If you have drugs, yeah, they'll listen. If not, they don't have time for you, basically.”

After describing some positive events in her life, Marie (32 years old) reflected on how this transactional support undercuts the positive progress she was experiencing in other areas of her life:

It's like, everything good is happening but then I'm just still stuck in this piece of shit area, where these girls out here act like they're your friend, but they're only your friend when you've got something.

Transactional types of support, or questionable motivations from others when providing help, were familiar to participants. Marie’s expressed self-reliance mirrors that of Jane being forced to learn self-reliance from a young age due to her mother’s substance use. Skepticism of others’ motivations behind offers of help was echoed to some extent by nearly all other participants and refers back to the reasons women felt they could not ask others for help.

Available support mainly came from people outside of the street economy, which was typically genuine support where nothing is expected in return. Marie (42 years old) demonstrates the in-group/out-group dichotomy of the street survival economy in a story about a woman who began supporting other WWUD-SS once she stopped using drugs:

Some of them do [help others], like the Black girl that was right there with the dog. She used to be out here. She used to be out here like I'm out here and everybody else was out here, but then she stopped. She still comes out here to make sure all of us is OK, and that's what I like. If I'm calling you my friend, don't only come around when it's beneficial to you. …She'll bring clothes out here for some of the girls that wear the same clothes for days. She'll take them to her hotel or wherever she is, so they can get in the shower. That's a true friend.

Family members were often cited as the most common source of genuine support. However, these relationships were affected by whether family members were involved in the street economy and therefore subject to the same economic instability, exposure to violence, and other socio-structural hardships. For example, Eve (43 years old) said she leans on the support of her sister and partner to deal with the challenges of her drug treatment program. However, Eve’s sister sets firm boundaries with her because of her history of stealing money to buy drugs. Nonetheless, Eve expressed a deep love and sense of caring between herself and her sister. Irene (41 years old) has a tumultuous relationship with her biological mother, who struggles with an addiction to crack cocaine and whose relationship more closely mirrors a transactional one as described above than genuine support.

Kylie (26 years old) does not have a perfect relationship with her mother—she described arguments between the two—but she described a close relationship rooted in unconditional support, underscoring the potential buffering effects of support from those from outside the street economy:

My family is extremely supportive of me. I talk to my mom almost every day. Basically, my mom mainly, she just tells me… "Whenever you're ready, just call me and you know, I'll get you."

### Limited formal resource utilization

Recent histories of formal resource utilization were nearly nonexistent in the interviews. In general, WWUD-SS had little experience with or knowledge of local resources to assist with physical or mental health issues outside of drug treatment or the emergency department. Examples of participants visiting a physician’s office, shelter, or non-profit for social services were infrequent and, when they occurred, had taken place months or years in the past; often participants could not remember the location or the name of the organization they visited (the exception was in the case of hospital or emergency department visits, as local hospitals are well known in Baltimore).

Lack of transportation and other logistical difficulties were the most commonly cited barriers to seeking assistance from formal resources. Women were most aware of resources that were within a few-block radius of the places where they lived, which alleviated the barrier of transportation but still did not guarantee use. These logistical barriers stemmed from structural inequities such as markers of economic marginalization including housing insecurity, lack of a reliable cell phone or internet connection to learn about resources or make appointments, and money for public transportation. One participant, Nia (30 years old) said the only reason she was able to seek out drug treatment was that the treatment center guaranteed her housing as part of their program.

Processes of social marginalization included anticipated or enacted stigma from care providers that left some women feeling as though help was barely in reach. The way Ann (38 years old) describes interactions with hospital staff in the following exchange shows that judgment of drug use is common and salient in the minds of WWUD-SS, and not just an issue she personally has experienced:

They judge you when you go in there [*the hospital]* like, "Oh, she's an addict. We are not going to give her nothing for the pain, nothing for withdrawals. Let her suffer." They judge you terribly. That's why nobody goes to the hospital around here. They get tired because they judge you. They're just snooty. "You're a user. Put her in room three." You hit the bell for the nurse and they never come. …Even knowing that might happen, it still was like, "My leg was in so much pain. I need to go."

Ultimately, Ann went to the hospital because of severe pain—but not without first second-guessing the potential helpfulness of going.

Participants also expressed a desire for mental health care, the majority of whom had received a formal mental illness diagnosis in the past and were keenly aware of their mental health needs. Often, participants had been diagnosed years prior as children or young adults but they had engaged with mental health care intermittently as adults, if at all. Rarely did participants express a recent history of mental health care sought out independently of the justice system or drug treatment.

The criminal legal system played a role in many women’s past diagnosis of mental illness and history of engagement in mental health care. For example, Helen knew that her anxiety was debilitating (“I knew I had something wrong with me”) but was not able to seek out professional care until she arrived in jail:

When I got locked up, they sat down. They talked to me. I guess the way I talked and didn't look 'em in the eyes, they said... They said that they can just tell just by lookin' at me and what I've been through and what I do and stuff like that can increase it [anxiety].

In fact, drug treatment, mental health care, and involvement with the criminal legal system were often inextricably intertwined for women. Court-ordered counseling was a typical route through which women received psychiatric or psychological care after their own arrest—typically in the context of court-ordered drug treatment.

### Children as motivation for resilience

Women with children often cited their children as their motivation for weathering life’s challenges. In some cases, their motivation was repairing broken relationships with their children or fostering fragile but intact ones. In other cases, children and grandchildren were a future-oriented lens through which women viewed their current difficulties. For example, Carly (44 years old) explained:

My motivation is watching my children grow into great mothers and fathers, women and men, adults. And watching my grandbabies grow into those great men and women. And I want to be there for every step because that's the joy they give me. It's joy there when it comes to those babies.

One comment from Marie (28 years old), who has young children, situated her needs with that of her children when it came to asking her former partner for money:

That would be selfish for me to call him and say, "Can you come drop $20 off because I don't feel good." No, because I look at my kids, they're the ones that need him. They cannot fend for themselves. He's their provider right now. I feel as it's not really taken from my kids, but it is taken from them because that $20 could go to them, going swimming, or going out to a movie, or something like that. I get out here and get it myself before I ask him for it.

### Coping, self-medication, and substance use

Substance use was often expressed as a distraction from traumatic experiences or the realities of a harsh environment, often one where social support and other external resources were perceived as unavailable. Multiple participants used the term “self-medicate” to describe their relationship to drug use and its function in response to their mental health. Self-medication or other means of coping through substance use are rational choices considering the significant social and structural barriers faced by study participants. For example, when asked how she was currently feeling, Georgia (35 years old) responded, “Lately, depressed. Very, very depressed. I feel like I just want to stay numbed. Yeah, just stay high. When I'm sober I'm freaking miserable.” Another example came from Helen (age 20):

I feel depressed when I'm sober. As soon as I get high, I’m happy. As soon as I'm sober, that's when everything gets me, which...I try not to let that happen, because I don't want to feel down.

Participants similarly described using drugs to numb psychological pain or blot out memories of traumatic experiences, albeit temporarily:

You numb yourself out. That's what it does to me. If I feel like I'm getting depressed, I'll do some dope and I'll be OK. It's self‑medicating. It works. The only time that I can honestly be like, ‘Yeah, I've gone through it [*experienced extreme hardship and emotional pain*],’ is when I got locked up because I didn't have nothing to turn to. (Marie, 32 years old)

Participants perceived their substance use as a coping mechanism for external stressors and trauma, though the efficacy and longevity of this “numbing” varied.

## Discussion

Among a sample of WWUD-SS in Baltimore, external resilience—as expressed as resource utilization and seeking social support—was extremely limited. External resilience was often stifled by immediate survival needs and transactional relationships that left women skeptical about the availability of genuine support available to them. Socio-structural factors, such as the criminalization of drugs and sex work, extreme poverty, and internal and external stigmas, underpinned participants’ difficulties in developing and harnessing external resilience. While qualities of internal resilience have heavily informed resilience literature, interventions, and, consequently, the public’s understanding of resilience, our findings show that only measuring internal resilience can be misleading and minimize the external forces shaping the concept. Our findings also show a critical need for a more comprehensive understanding of resilience, particularly with marginalized populations such as WWUD-SS, as well as greater precision in the language of resilience to better inform research and intervention [47].

WWUD-SS’ ability to develop and utilize external resilience appears to be undercut through two different pathways. The first is through experiencing multiple structural vulnerabilities, driving competition for resources from social networks, and forming the basis of participants’ descriptions of pervasive transactional-type relationships. As participants described, transactional relationships from peers tended to diminish the perceived value of support from others, leaving women resigned to face hardships alone. Familial relationships and their strength of support appeared to be related to the family member’s own history of substance use: In cases where a family member also used drugs or sold sex, participants tended to describe these relationships as more like peers (i.e., transactional). This finding echoes what Padgett et al*.* describe as “good news/bad news” with family members of individuals experiencing homelessness and substance abuse [52]. For these individuals, as with many women in the current study, familial relationships were fluid and constantly evolving: Family members may be sources of support during difficult times but often struggled with their own substance use or other chronic life stressors that “thinned the ranks of family networks” and their ability to provide support [52]. In recent years, researchers and academics began to integrate social isolation from the larger society into multidimensional definitions of poverty [53]. Yet our results show that the social isolation expressed by WWUD-SS need not only be from outside this population, but also contributes to isolation within the population. Further, realized but unhelpful social support from peers and family places WWUD-SS at risk of exacerbating social isolation and psychological distress, distinct from their male counterparts [41].

A second pathway through which WWUD-SS external resilience is limited is through the dual social stigmatization of drug use and sex work. Participants described reluctance to ask others for help after violence or when experiencing drug withdrawal—despite these being both physically and psychologically painful—because they believed they deserved negative consequences and therefore were undeserving of support. Participants’ internalized stigma of their drug use and sex work left them feeling undeserving of support and therefore unable to consider asking for social or organizational support from others. Study participants also described how past incidents of enacted stigma, predominantly from healthcare professionals, created feelings of anticipated stigma when considering future engagement with resources. WWUD-SS in the present study expressed times where they did access health or social services, only to be treated poorly or their needs ignored. Often, these experiences precluded them from readily seeking out resources in the future despite the expressed need. Shame, self-blame, and general unworthiness of help that participants described are informed by WWUD-SS’ collective experiences of social and structural inattention or incompetence (e.g., sub-optimal health care, police inattention to their victimization), shaping their expectations about the treatment they will receive from others outside of the community [21, 55]. Broad negative social attitudes about substance abuse, sex work, and experiencing poverty namely that these are personal choices (or consequences of personal choices) requiring personal responsibility for “fixing” problems further reinforce the perception that support is wholly unavailable [54].

Both of these potential pathways not only shape external resilience but also incorporate context from socio-structural factors that are typically absent from understandings of internal resilience among WWUD-SS. Many women said they did not want or need help from others—but this should not be confused with internal resilience in the traditional sense: WWUD-SS often expressed resignation that they had no other option but to tackle problems on their own. To be sure, WWUD-SS’ survival in the face of extreme difficulty is indeed a facet of resilience and speaks to participants’ strength. However, qualities that have been conceptualized and measured as markers of internal resilience are not necessarily applicable for substance-using populations such as this sample of WWUD-SS, considering the stated reliance on emotional numbing and the difficulty of existing measures to distinguish between resilience and resignation to self-reliance. For example, “bouncing back” after hardship is one common marker of internal resilience, with the implication being that bouncing back does not include substance use to aid the process. In fact, studies often use substance use as an outcome indicating *poor* internal resilience [47]. However, our results echo other research that shows a more complex relationship between resilience and substance use.

Improving access to resources is a critical step in strengthening external resilience and ultimately improving mental health. In this sample, perceived availability and actual access to local health and social service resources are extremely limited despite the expressed need for these supportive services. Interviews with WWUD-SS also corroborated many prior findings about cost and transportation, lack of knowledge of resources, and conflicting priorities with drug use being significant barriers to accessing services [48, 56]. These findings highlight the critical need for services that are welcoming, non-stigmatizing, flexible, and holistic. As we and others have written about previously, colocating services commonly needed by WWUD-SS (e.g., syringe services, counseling, and HIV testing and care) can alleviate some of these barriers and empower these populations to access services in times of need [56]. Additionally, peer mentoring or guidance on formal resources for health and social services can be a useful way to promote resilience and social support within the same intervention [57]. Yet improving access to resources also includes improving access to *quality* care. Too often, WWUD-SS face explicit stigma from health providers about their engagement in illicit behaviors or otherwise receive poorer quality of care than women who do not engage in these behaviors. As stated above, poor treatment due to substance use or sex work can be highly stigmatizing and alienate WWUD-SS from future resource engagement. Our findings speak to a need for increased structural and cultural competence related to substance use and sex work engagement on the part of providers to establish trusted and high-quality sources of care for WWUD-SS [55, 58, 59].

WWUD-SS employed logical and practical coping mechanisms in the face of these harsh realities including through drug use. Drug use is often a double-edged sword for women: It provides immediate relief in the face of struggles when faced with limited other options, but also creates a barrier to social and other sources of external support. WWUD-SS face a tension between drug use as a way to cope and drug use as a factor that partially shapes the transactional relationships driven by competition for money and drugs when economic resources are thin. Empowerment interventions have shown promising results in building a sense of community cohesion and generating community mobilization for WWUD-SS around the world [60]. Empowerment interventions, however, do not fully address the complex role that drug use plays in creating competition for survival in the absence of other ways of making money beyond sex work. Economic interventions such as guaranteed basic income or microfinance are often not considered domains of public health, but our results show one potential pathway through which economic empowerment can improve social support and, consequently, resilience. Individual- and community-level socioeconomic inequities in education, employment, and neighborhood development have all been associated with both greater exposure to trauma and diminished internal and external resilience [61, 62]. Research and programmatic focus on internal resilience and its improvement in the absence of social justice considerations—including access to economic and health-related resources—will continue to show a misleading picture of resilience and may even be harmful to marginalized populations such as WWUD-SS who may be blamed for lacking resilience despite facing great structural hurdles to its improvement.

These findings should be considered in light of the study limitations. First, the interviews are subject to potential social desirability bias and other limits of self-reporting. Second, we recruited the sample for this study (and the parent study, EMERALD) using street-based methods. Though the eligibility criteria did not specify street-only sex work, the recruitment method may have skewed the sample toward the most structurally vulnerable women. As such, these results should also not be considered representative of all WWUD-SS and may have limited generalizability outside of the context of Baltimore City. Resilience is, in part, a culturally bound construct; manifestations of social support or relevance of particular resources being utilized will likely differ by local context and more research is needed to replicate these findings across cultures. However, our findings of socio-structural barriers to accessing resources are similar to other published reports of WWUD-SS in other locations and provide a basis with which to conduct further research in other geographic settings [48].

Another limitation to consider is the positionality of the interviewers and coders, i.e., how one’s social identities inform how they see the world and how the world sees them. First, there were no Black interviewers or coders despite half the participants identifying as Black. Second, no interviewer or coder had lived experience as either a sex worker, a person who uses drugs, or was currently experiencing severe economic insecurity. As such, differing backgrounds likely shaped the authors’ interpretations of participants’ narratives. While the interviewers were all experienced in qualitative methods and worked with WWUD-SS for several years, this lack of lived experience and affiliation with a powerful local institution may have caused participants to be reticent to share details about their engagement in illegal activities for fear of researchers’ disclosure to police or poor treatment toward the participant (despite reassurances in the consent process). Further, participants’ familiarity with the University and Johns Hopkins Hospital (JHH) may predispose them to respond in ways they believe are expected of them by the researchers, in particular pro-health or anti-substance use messages. For example, it is possible that participants mentioned their histories of drug treatment because they felt that was expected of them by the researchers. One participant mentioned twice in her interview that she “loved” JHH; when asked about any drawbacks to seeking care at JHH she said she could not think of any. This may be true, but it is worth reflecting on the role that the researchers’ affiliation may have had on her answer.

## Conclusions

Our findings provide new insight into the limitations of exclusive focus on internal resilience, particularly among people who use drugs, experience structural vulnerabilities, and face significant socio-structural threats to their ability to strengthen external resilience. Improving connections to community resources is a targeted and potentially impactful way to strengthen external resilience in this population. Wider policy changes that reduce structural vulnerabilities among WWUD-SS—such as broader access to safe and affordable housing or new economic opportunities—may create an environment in which women can build internal and external resilience.

## Data Availability

The datasets generated and/or analyzed during the current study are not publicly available due to participant confidentiality protections but are available from the corresponding author on reasonable request.
